# TTP-like syndrome: novel concept and molecular pathogenesis of endotheliopathy-associated vascular microthrombotic disease

**DOI:** 10.1186/s12959-018-0174-4

**Published:** 2018-08-11

**Authors:** Jae C. Chang

**Affiliations:** Department of Medicine, University of California Irvine School of Medicine, Irvine, CA USA

**Keywords:** ADAMTS13, Complement, Disseminated intravascular coagulation (DIC), Disseminated intravascular microthrombosis (DIT), Endotheliopathy, Microthrombogenesis, Thrombotic thrombocytopenic purpura (TTP), TTP-like syndrome, Unusually large von Willbrand factor multimers (ULVWF), Vascular microthrombotic disease (VMTD)

## Abstract

TTP is characterized by microangiopathic hemolytic anemia and thrombocytopenia associated with brain and kidney dysfunction. It occurs due to ADAMTS13 deficiency. TTP-like syndrome occurs in critically ill patients with the similar hematologic changes and additional organ dysfunction syndromes. Vascular microthrombotic disease (VMTD) includes both TTP and TTP-like syndrome because their underlying pathology is the same disseminated intravascular microthrombosis (DIT). Microthrombi are composed of platelet-unusually large von Willebrand factor multimers (ULVWF) complexes. TTP occurs as a result of accumulation of circulating ULVWF secondary to ADAMTS13 deficiency. This protease deficiency triggers microthrombogenesis, leading to “microthrombi” formation in microcirculation. Unlike TTP, TTP-like syndrome occurs in critical illnesses due to complement activation. Terminal C5b-9 complex causes channel formation to endothelial membrane, leading to endotheliopathy, which activates two different molecular pathways (i.e., inflammatory and microthrombotic). Activation of inflammatory pathway triggers inflammation. Activation of microthrombotic pathway promotes platelet activation and excessive endothelial exocytosis of ULVWF from endothelial cells (ECs). Overexpressed and uncleaved ULVWF become anchored to ECs as long elongated strings to recruit activated platelets, and assemble “microthrombi”. In TTP, circulating microthrombi typically be lodged in microvasculature of the brain and kidney, but in TTP-like syndrome, microthrombi anchored to ECs of organs such as the lungs and liver as well as the brain and kidneys, leading to multiorgan dysfunction syndrome. TTP occurs as hereditary or autoimmune disease and is the phenotype of ADAMTS13 deficiency-associated VMTD. But TTP-like syndrome is hemostatic disorder occurring in critical illnesses and is the phenotype of endotheliopathy-associated VMTD. Thus, this author’s contention is TTP and TTP-like syndrome are two distinctly different disorders with dissimilar underlying pathology and pathogenesis.

## Background

The new term vascular microthrombotic disease (VMTD) has been used [1–3], but has not been designated as a disease entity yet. Both microvascular thrombosis and vascular microthrombosis have been used interchangeably to describe the similar pathological conditions. Disseminated VMTD represents both TTP and TTP-like syndrome, but the identity of TTP-like syndrome has not been clearly defined to date.

TTP is characterized by microvascular thrombosis associated with markedly decreased ADAMTS13 activity due to mutation of ADAMTS13 gene (hereditary) or due to antibody against ADAMTS13 (acquired). Hematologic phenotype is consumptive thrombocytopenia and microangiopathic hemolytic anemia (MAHA) as a result of disseminated intravascular microthrombosis (DIT) [2, 4]. Organ involvement occurs typically in the brain and kidneys. DIT is the pathological condition caused by vascular “microthrombi”, which are exclusively composed of the complexes of platelet and unusually large von Willbrand factor multimers (ULVWF) in various organs [4–6]. In 1924, Eli Moschcowitz first recognized a thrombotic blood disorder characterized by disseminated hyaline microthrombi in terminal arterioles and capillaries of organs in a young woman who died at the Beth Israel Hospital in New York City [7]. Later, Singer et al. named this disorder thrombotic thrombocytopenic purpura (TTP) [8].

Other focal, multifocal or localized microvascular thrombosis also exists without proper name designation. Appropriate medical term could be focal, multifocal or localized VMTD. These may include ischemic stroke syndrome (e.g., transient ischemic attack) [9, 10] and myocardial ischemia (e.g., angina) [10]. Also, focal endotheliopathy in HERNS disease and Susac syndrome as well as localized endotheliopathy in Kasabach-Merritt syndrome are suspected to be due to microthrombosis in smaller or larger vasculatures. These syndromes cannot be designated as TTP because their involvement in the organs is not generalized and is usually not associated with thrombocytopenia and MAHA.

More recently, TTP-like syndrome has been frequently reported. It is also characterized by vascular microthrombosis with thrombocytopenia and MAHA, but commonly atypical organ dysfunction syndromes such as acute respiratory distress syndrome (ARDS), rhabdomyolysis, acute fulminating hepatic failure, and pancreatitis have occurred. These syndromes are also characterized by DIT [1–3]. Therefore, to properly classify TTP-like syndrome in the category of VMTD, there is a need for differentiating between TTP and acquired TTP-like syndrome and identifying the pathogenesis of TTP-like syndrome since it is not associated with ADAMTS13 antibody.

### TTP-like syndrome

TTP typically involves the brain and kidneys, but TTP-like syndrome prominently develops in one or more of vital organs [11–14], including the liver [15, 16], heart [17, 18], lungs [11–13, 19, 20], pancreas [21] and others with or without involvement of the brain and kidneys. Oftentimes fewer schistocytes are present on the blood film and intravascular hemolysis could have easily missed [11, 13]. Since MAHA is not overt because of fewer schistocytes, the diagnosis of TTP-like syndrome could have been masked even though thrombocytopenia was present [2]. Because of less prominent nature of schistocytes, it has been termed atypical MAHA (aMAHA) [1, 2]. The most notable observation of TTP-like syndrome is its frequent occurrence in critical illnesses such as infection, sepsis, trauma, cancer, autoimmune disease, malignant hypertension, drug and toxin, envenomation, and complications of pregnancy, surgery and transplant (Table 1).

**Table 1:** Genesis and characteristics of VMTD in TTP and TTP-like syndrome

	Hereditary TTP (GA-VMTD) Acquired TTP (AA-VMTD)	TTP-like syndrome (EA-VMTD)
Primary causes/events	Hereditary ADAMTS13 gene mutation Acquired ADAMTS13 antibody formation ↓	Pathogen (e.g., viruses; bacteria; fungi; rickettsia; parasites) Polytrauma (e.g., chest/lung; bone; skull/brain injury) Pregnancy (e.g., preeclampsia; abruptio placenta; amniotic fluid embolism) Cancer (e.g., disseminated stomach/breast/lung cancer) Transplant (e.g., liver; kidney; bone marrow) Drug and toxin (e.g., cyclosporine; mitomycin C; Shiga toxin) ↓
Secondary event	Excessive circulating mULVWF ↓	Complement activation (C5b-9) and endothelial injury → endotheliopathy ↓
Tertiary event	Microthrombogenesis → platelet-ULVWF complexes ↓ Microthrombi lodged in arteriolar and capillary lumens ↓	Cytokine release → inflammation → SIRS Platelet activation and endothelial exocytosis of eULVWF ↓ Microthrombogenesis → platelet-ULVWF complex strings ↓
Final event	Microvascular microthrombosis ↓ DIT/VMTD ↓ TTP	Vascular microthrombosis ↓ DIT/VMTD ↓ TTP-like syndrome
Hematologic features		
Platelet	Consumptive thrombocytopenia	Consumptive thrombocytopenia
Red blood cell	MAHA	MAHA/aMAHA
Clinical syndromes		
Inflammation	Uncommon	Very common
Cytokine storm	Absent	Often present in sepsis and MODS
SIRS	Absent	Often present in sepsis and MODS
Encephalopathy	Very common	Common, especially in HUS
ARDS	Probably absent	Common
AFHF	Probably absent	Common, sometimes with hepatic coagulopathy
ARF/HUS	Very common	Common
“DIC” (see text)	Doesn’t occur	Identical to TTP-like syndrome
Laboratory features		
ADAMTS13 activity	Markedly decreased (< 5% of normal)	Mild to moderately decreased (20–70% of normal)
ADAMTS13 antibody	Positive in acquired TTP	Negative
Haptoglobin	Markedly decreased	Markedly decreased
Schistocytes	++ to ++++	None to +++
Therapeutic response to		
TPE	Very good response	Excellent and fast response if treated in early stage
Platelet transfusion	Contraindicated	Contraindicated
rADAMTS13	Unknown; expected to be effective in GA-VMTD	Unknown; expected to be very effective

*AFHF* acute fulminant hepatic failure, *ARF/HUS* acute renal failure/hemolytic uremic syndrome, *ARDS* acute respiratory distress syndrome, “*DIC*” disseminated intravascular coagulation of McKay, *ECs*, endothelial cells, *eULVWF/mULVWF* endothelial unusually large von Willebrand factor/megakaryocytic ULVWF, *LDH* lactate dehydrogenase, *MAHA/aMAHA* microangiopathic hemolytic anemia/atypical MAHA, *rADAMTS13* recombinant ADAMTS13, *SIRS*, systemic inflammatory response syndrome, *TMA* thrombotic microangiopathy *TPE*, therapeutic plasma exchange; TTP, thrombotic thrombocytopenic purpura, *VMTD* vascular microthrombotic disease

In the early 1980s, Moake et al. made a very important discovery that ULVWF contributed to the pathogenesis of TTP [22]. Furlan et al. [23] and Tsai [24] independently published the presence of VWF-cleaving protease, and subsequently the deficient role of this protease ADAMTS13 due to anti-ADAMTS13 antibody was established.

Prior to the role of autoantibody was recognized, clinicians accepted the use of the term TTP whenever a patient presented with the dyad of thrombocytopenia and MAHA, even though obvious organ dysfunction was not present yet [12]. This dyadic feature was considered to be sufficient criteria to make the diagnosis of TTP for the purpose of initiating urgent therapeutic plasma exchange (TPE) to save lives [11–13]. The generic term TTP, encompassing both TTP and TTP-like syndrome, has served well for the patient by allowing TPE when presented with thrombocytopenia and MAHA/aMAHA even though organ dysfunction is not developed yet. TPE has been very effective and life-saving measure in both disorders when it was employed in the earliest possible time [2, 11–13, 20].

Because of common occurrence of TTP with acute renal failure/hemolytic-uremic syndrome (HUS) in clinical medicine, the combined term TTP-HUS also has been in use to include both TTP and TTP-like syndrome to date [25] even though the pathogenesis and clinical features of HUS are clearly different from TTP [3]. In retrospect, this combined term might have contributed to the masking of TTP-like syndrome and VMTD when organ dysfunction developed in other than the brain and kidneys. It also has delayed identifying the multifaceted pathogenesis of TTP-like syndromes. In addition, this terminology could have kept disseminated intravascular coagulation (“DIC”) as a different disease from TTP-like syndrome [26]. Quotation marks have been placed on “DIC” to note that it is different from true DIC, which causes fibrin clots composed of fibrin meshes that is seen in acute promyelocytic leukemia (APL).

Hematologists have been puzzled when encountered acquired TTP-like syndrome with negative ADAMTS13 antibody and phenotype of thrombocytopenia and MAHA. This syndrome has occurred with atypical organ phenotypes. Such syndromes include the hemolysis, elevated liver enzymes and low platelet (HELLP) syndrome [16], acute respiratory distress syndrome (ARDS) [2, 13, 19, 20], HUS [3, 27, 28], acute myocardial infraction [17, 29, 30], acute pancreatitis [21, 31, 32], rhabdomyolysis [33, 34], encephalopathy [35, 36], viral hemorrhagic fevers [1, 37–40] and many others [2, 12–14].

### TTP vs. TTP-like syndrome

TTP and TTP-like syndrome are characterized by hematologic phenotypes of VMTD presenting with consumptive thrombocytopenia and MAHA. TTP occurs in two conditions: one is gene mutation-associated VMTD (GA-VMTD) and the other is antibody-associated VMTD (AA-VMTD). GA-VMTD, known as Upshaw-Schulman syndrome, is the result of homozygous or compound heterozygous mutations of ADAMTS13 gene. However, AA-VMTD is autoimmune disease resulting from ADAMTS13 antibody.

In contrast, TTP-like syndrome develops due to endotheliopathy-associated VMTD (EA-VMTD) in critical illnesses such as sepsis and trauma [2, 16, 40–43] as illustrated in Table 1. The pathologic nature of microthrombi, which are composed of platelet and ULVWF complexes, are the same in both TTP and TTP-like syndrome [2–6]. However, the pathophysiological mechanism forming microthrombi appears to be different, and in TTP, their physical configuration in vivo is not clearly defined at this time. In TTP-like syndrome, the organ localization of microthrombi is distinctly different among different organs and within the same organ; perhaps it is due to endothelial heterogeneity and organotropism [3]. To annotate the clinical and organ dysfunction syndromes, occurring as a result of endotheliopathy in critical illnesses, a novel “two-activation theory of the endothelium” has been proposed [1, 2, 26].

### Pathogenesis of TTP-like syndrome

#### Thrombocytopenia in critically ill patients (TCIP)

The earliest suspicion of TTP-like syndrome should come from unexplained thrombocytopenia in the critically ill patient. After exclusion of known causes of thrombocytopenia such as heparin-induced, drug or transfusion-related, consumptive coagulopathy-associated and hypersplenism-caused thrombocytopenia, and others [2], the term TCIP has been used to identify etiology-undetermined thrombocytopenia in critically ill patients. It is particularly well known in infectious diseases, including bacterial, viral, rickettsial, fungal and parasitic sepsis, are associated with TCIP [37–41, 44, 45]. It also occurs in non-infectious illnesses (e.g., severe trauma, cancer, complications of surgery, pregnancy and transplant, and immunologic and collagen vascular diseases) [2, 13, 42, 43, 46, 47].

Recently, significant correlation has been noted between the degree of thrombocytopenia, and severity and outcome of critical illnesses [48, 49]. Severer thrombocytopenia has been associated with systemic inflammatory response syndrome (SIRS) and multiorgan dysfunction syndrome (MODS) [50, 51]. These observations support TCIP is a key participant in the pathogenesis of critical illnesses, leading to VMTD. Now it is clear that TCIP is consumptive thrombocytopenia in the process of DIT in critically ill patients [1–3].

#### Role of complement activation on the endothelium

The activation of complement system is one of the pivotal events in innate immune defense mechanism of the host against pathogen. Its protective function for the host is to detect and eliminate invading microorganisms. Opsonization of foreign surfaces by covalently attached C3b fulfills three major functions: cell clearance by phagocytosis; amplification of complement activation by the formation of surface-bound C3 convertase; and assembly of C5 convertases [51]. Following activation of complement system through one of three pathways (classical, alternative, and lectin), cleavage of C5 induces the formation of multi-protein pore complex (C5b-9) (i.e., membrane-attack complex [MAC]), which leads to cell lysis.

However, despite its protective role for the host, when complement system becomes activated in critical illnesses [52, 53], C5b-9 also can attack innocent bystander host endothelial cells (ECs). If CD59 glycoprotein expressed on the endothelial cells [3] is downregulated due to critical illnesses, channel (pore) formation occurs in the endothelial membrane, which leads to endotheliopathy and even endothelial membrane lysis [53, 54]. Although the “imbalanced”, “uncontrolled” or “dysregulated” complement activation has been implicated to be the mechanism of atypical HUS, perhaps “unprotected” endothelium due to loss of CD59 protective effect against C5b-9 could be the mechanism leading to endotheliopathy, not only in atypical HUS [53], but also in the critical illnesses.

It is interesting to note that congenital CD59 deficiency due to its gene mutation has been associated with thrombosis and hemolytic anemia [55], which also suggest the close relationship between endothelial CD-59 loss and endotheliopathy. The study of the role of CD59 in the pathogenesis of endotheliopathy is needed.

#### TTP-like syndrome in the critical illness

TTP-like syndrome typically occurs in critically ill patients [1, 2, 12–14, 17–21, 25–50, 56]. Inexplicably, in current clinical practice and medical literature, the most of the patients with critical illnesses presenting with VMTD with or without hemorrhagic disorder have been identified as having “DIC” with either compensated (chronic) or decompensated (acute) designation [26]. It should be emphasized that “DIC” mimics TTP-like syndrome [1] and chronic DIC is identical to TTP-like syndrome in precipitating factors (i.e., critical illnesses), pathological findings (i.e., hyaline microthrombi), and hematologic features (i.e., thrombocytopenia) [26]. Later, reinterpretation of “DIC” will be separately discussed in more detail.

#### “Two-activation theory of the endothelium”

**Fig. 1:**
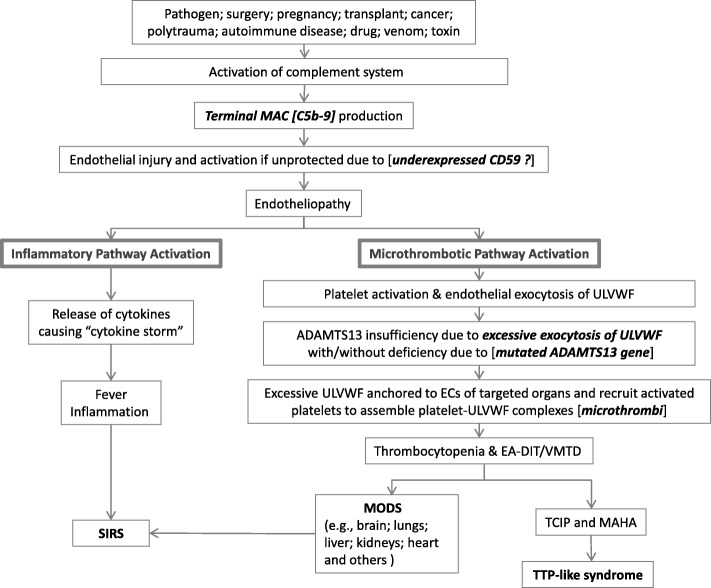
Molecular pathogenesis of TTP-like syndrome Fig. 1 elaborates “two activation theory of the endothelium”, which shows complement-induced endothelial molecular events, leading to endotheliopathy-associated DIT (i.e., TTP-like syndrome) and MODS. The organ phenotype syndrome in MODS includes encephalopathy, ARDS, AFHF, ARF/HUS, MI, AI, pancreatitis, rhabdomyolysis, "DIC", HELLPs, SS, and others. For example, in sepsis complement activation is the initial critical event. Complement activation can occur through one of three different pathways (i.e., classical, alternate and lectin). In addition to lysis of pathogen by terminal product C5b-9, it could induce endotheliopathy to the innocent bystander ECs of the host. C5b-9-induced endotheliopathy is suspected to occur if the endothelium is “unprotected” by CD59. Activated inflammatory pathway provokes inflammation in sepsis, but inflammation could be modest if the number of organ involvement is limited. Activated microthrombotic pathway results in endotheliopathy-associated DIT if the excess of ULVWF develops following endothelial exocytosis as a result of relative insufficiency of ADAMTS13 with/without mild to moderate ADAMTS13 deficiency, which is associated with heterozygous gene mutation or polymorphism of the gene. This theory explains all the manifestations of VMTD as illustrated in the Fig. 1. Abbreviations: AFHF, acute fulminant hepatic failure; AI, adrenal insufficiency; ARDS, acute respiratory distress syndrome; ARF, acute renal failure; “DIC”, false disseminated intravascular coagulation; DIT, disseminated intravascular microthrombosis; ECs, endothelial cells; EA-DIT, endotheliopathy-associated DIT; HELLPs, hemolysis, elevated liver enzymes, and low platelet syndrome; HUS, hemolytic uremic syndrome; MI, myocardial infarction; MODS, multi-organ dysfunction syndrome; MAHA, microangiopathic hemolytic anemia; SIRS, systemic inflammatory response syndrome; SS, stroke syndrome; TCIP, thrombocytopenia in critically ill patient; TTP, thrombotic thrombocytopenic purpura; ULVWF, unusually large von Willebrand factor; VMTD, vascular microthrombotic disease

Activated complement system provides a critical and multifaceted defense against infection, but it can be also activated in non-pathogen-induced critical illnesses such as polytrauma, pregnancy, surgery, transplant, autoimmune disease, and cancer [57–61]. Following the activation, complement can clear invading microorganisms by lysis or opsonization [27]. However, on the host side, the complement activation product terminal C5b-9 also could attack the host ECs and cause transmembrane channel formation on the endothelium and induce endotheliopathy. In turn, endotheliopathy triggers multiple molecular events [1–3, 55] as presented in Fig. 1.

Critical illnesses are well known to cause an injury to ECs, leading to endotheliopathy and endothelial dysfunction [62, 63]. Now, the evidence shows that complement activation plays the major role in molecular pathogenesis of inflammation and DIT [1–3, 55, 58–61, 64]. Based on endotheliopathy that promotes several biomolecular events, the “two-activation theory of the endothelium” is proposed and illustrated in Fig. 1 [1, 2]. Endotheliopathy triggers the activation of two independent endothelial pathways (i.e., inflammatory and microthrombotic). In short, two important molecular events are: 1) release of inflammatory cytokines (e.g., interleukin [IL]-1, IL-6, tumor necrosis factor-α, and others) [63, 65, 66], and 2) activation of the platelet [67] and endothelial exocytosis of ULVWF [68, 69]. The former initiates inflammation through “activation of inflammatory pathway”, and the latter mediates microthrombogenesis via “activation of microthrombotic pathway” [1–3, 26] as shown in Fig. 1.

In endotheliopathy, microthrombogenesis is a process, in which long elongated ULVWF strings are anchored to ECs after release from Weibel-Palade bodies and recruit platelets, promoting the formation of platelet-ULVWF complexes [5, 6, 70]. These microthrombi strings are the pathologic complexes formed, perhaps under the shear stress of blood flow, leading to endotheliopathy-associated DIT and hematologic features of TTP-like syndrome [2, 4].

### Microthrombogenesis in VMTD

#### Megakaryocytic ULVWF(mULVWF) and endothelial ULVWF(eULVWF)

Two kinds of ULVWF are synthesized in two different sites (i.e., megakaryocytes and ECs) as shown in Table 2 [4, 71]. Megakaryocytic ULVWF (mULVWF) are released normally into circulation as platelet-adherent form and stored in α granules of the platelet. But endothelial ULVWF (eULVWF) are produced in ECs and stored in Weibel-Palade bodies to be available as endothelium-adherent form following release from ECs to initiate normal hemostasis in vascular injury [4, 6, 72].

**Table 2:** Characteristics of two different ULVWF multimers

	mULVWF multimers	eULVWF multimers
Synthesized in	Megakaryocytes	Endothelial cells
Stored in	α granules of platelets	Weibel-Palade bodies of ECs
Primary distribution at release	In circulation	On the membrane of ECs
Availability	In microcirculation	At ECs following endothelial exocytosis
Exposure to ADAMTS13	As platelet-adherent form	As ECs-adherent form
Interaction with platelets causing	Platelet aggregation and adhesion	Platelet-ULVWF strings
Localization of platelet-ULVWF complexes	Arteriolar and capillary lumens lodged as microthrombi *in situ*	Endothelial membrane-anchored as microthrombi strings
Example of leading its activity	ADAMTS13 autoantibody	Sepsis-induced endotheliopathy
Endotheliopathic lesion	Microthrombotic microangiopathy	Microthrombotic angiopathy
Hematologic manifestation	Thrombocytopenia and MAHA	Thrombocytopenia and MAHA/aMAHA
Associated inflammation	None to minimal (?)	Mild to severe
Associated clinical syndrome	TTP	TTP-like syndrome

*ECs* endothelial cells, *eULVWF/mULVWF* endothelial unusually large von Willebrand factor/megakaryocytic ULVWF, *MAHA/aMAHA* microangiopathic hemolytic anemia/atypical MAHA, *TTP* thrombotic thrombocytopenic purpura

Although the differences in structure and function between mULVWF and eULVWF are unknown at this time, it appears two different ULVWF multimers from different origin may have different functions [73, 74]. Microthrombogenesis of TTP occurs in microcirculation [4, 75] due to hyperactivity of circulating mULVWF in hereditary and antibody-associated ADAMTS13 deficiency. However, TTP-like syndrome is likely associated with relative insufficiency of ADAMTS13 as a result of excessive exocytosis of eULVWF in endotheliopathy [2]. In TTP, mULVWF multimers might react with platelets and assemble microthrombi in the microvasculature *in situ* under the shear stress, but in TTP-like syndrome eULVWF strings are anchored to ECs and get decorated with platelets to form microthrombi strings [5, 6, 70].

In severe sepsis, decreased ADAMTS13 activity is correlated with greater adhesion capacity of ULVWF and higher degree of thrombocytopenia as well as severity of critical illnesses and organ dysfunction [76]. Decreased ADAMTS13 activity in some patients with TTP-like syndrome also suggests underling partial ADAMTS13 deficiency could exist as well. In addition to endothelial exocytosis of ULVWF, partial ADAMTS13 deficiency associated with polymorphism or heterozygous mutation of the gene could contribute to the onset and degree of severity of TTP-like syndrome [77–80].

Both ULVWF lodged in capillaries and ULVWF anchored to ECs are rapidly cleaved by ADAMTS13 in vitro [4, 6, 72]. This observation is certainly consistent with the benefit of TPE for TTP due to ADAMTS13 deficiency and TTP-like syndrome with its insufficiency.

#### Dissimilarity between TTP and TTP-like syndrome

The dissimilar pathogenesis and different phenotypic characteristics between TTP and TTP-like syndrome are summarized in Table 1. It is hypothesized that microthrombogenesis in acquired TTP is caused by hyperactivity of mULVWF due to anti-ADAMTS13 antibody and occurs in the microvasculature *in situ*. On the other hand, microthrombogenesis in TTP-like syndrome is triggered by excessive exocytosis of eULVWF from ECs and occurs in the endothelial membrane. In both cases, deficient and or insufficient ADAMTS13 could not handle the excess of ULVWF.

This slightly different microthrombogenesis could lead to different organ localization and configuration of microthrombi, but still produce the same hematologic phenotype of VMTD. In TTP, microthrombi formed in the microvasculature become lodged within arterioles and capillaries of the brain and kidneys [4], which condition can be called “microvascular” microthrombosis. In TTP-like syndrome, it takes place on the smaller and larger vasculatures [80–83] of various organs depending upon endothelial heterogeneity that determines organ localization [83–85], which condition could be called “vascular” microthrombosis. We know more about endothelial microthrombogenesis in TTP-like syndrome, but do not know how microthrombogenesis in TTP occurs in microcirculation *in vivo* other than that it might be promoted under the condition of shear stress due to blood flow.

Without the understanding the endothelial molecular pathogenesis of TTP-like syndrome, clinicians have thought the atypical feature of different organ involvement in TTP-like syndrome is just a variant of TTP. In reality, different organ phenotypic TTP-like syndromes occur as a result of endothelial heterogeneity caused by genetic variables [3, 83–85] through endowed molecules in ECs (e.g., CD59 and Gb3 in HUS) [3, 86]. For examples, extra renal manifestations of Shiga toxin-producing *E. coli*-HUS (STEC-HUS) represent expression of endothelial heterogeneity caused by endowed molecules, localizing in the brain with encephalopathy, heart with myocardial infarction, pancreas with pancreatitis, and others [3].

### Reinterpretation of “DIC”

**“**DIC” has been the most intriguing disease among all the human diseases because of its deadly nature and conundrums as listed follows [26]:

- No clearly defined clinical and pathological diagnostic criteria are available.
- Not a single test or set of the tests can confirm and establish the diagnosis.
- Unexplained bleeding disorders such as viral hemorrhagic fevers are often blamed to it without foundation.
- Establishing the diagnostic application has been very subjective among investigators.
- The scoring system for the diagnosis is imprecise, confusing and subjective.
- The pathogenesis (i.e., tissue factor [TF]-FVIIa activated coagulopathy) has never been proven.
- Not a single treatment has been clearly proven to be effective.
- No therapeutic benefit has occurred even after numerous clinical trials.

Only consistent clinical, pathologic and hematologic features are:

- It occurs in critical illnesses (e.g., sepsis) and APL, but with different phenotypes.
- Clinical features are VMTD (i.e., DIT).
- Pathologic features are arteriolar and capillary hyaline microthrombi.
- Hematologic features are thrombocytopenia and MAHA.

True DIC (e.g., consumption coagulopathy), which occurs in APL [87, 88], is a coagulation (hemorrhagic) disorder, developing due to activation of TF-FVIIa complex-initiated coagulation cascade. In APL, TF is strongly expressed in leukemic promyelocytic cells. TF triggers fibrinogenesis via activation of FVII. On the other hand, “DIC”, which occurs in critical illnesses, has also been named as DIC based on the same TF-initiated coagulation (thrombotic) disorder [89, 90]. However, the clinical and hematologic features are very different between APL and critical illnesses. Instead, the clinical, pathological and hematological features of “DIC” are identical to endotheliopathy-associated DIT [1, 2], which is microthrombotic disorder. The differences between “DIC” (i.e., microthrombi) and true DIC (i.e., fibrin clots) are summarized in Table 3.

**Table 3:** Hematologic and Clinical Characteristics of endotheliopathy-associated DIT and true DIC

	EA-DIT/VMTD and “DIC” of McKay	True DIC
Example	TTP-like syndrome	APL
Nature of the clots	“Microthrombi strings” made of platelet-ULVWF complexes	“Fibrin clots” made of fibrin meshes
Mechanism of the genesis	Intravascular microthrombogenesis	Intravascular fibrinogenesis
Inciting causes/events	Infection; surgery; pregnancy; transplant; cancer; drug; toxin, leading to edotheliopathy	APL, leading to TF expression
Hematological manifestation	Microthrombotic disorder	Hemorrhagic disorder
Pathogenesis		
Mechanism	Activation of microthrombotic pathway	Activation of TF-initiated coagulation cascade
Site of activation	Intravascular membrane of ECs	In circulation
Thrombopathic result	Intravascular hemostasis of ULVWF path	Consumption of fibrinogen, FV and FVIII
Effect on the involved organ	Hypoxic organ dysfunction	Generalized bleeding tendency
Coagulation tests		
Fibrinogen	Normal	Decreased
PT; aPTT; TT	Normal	Prolonged
FVIII activity	Normal or markedly increased	Markedly decreased
Thrombocytopenia	Mild to moderately severe	Not consumed but decreased due to APL
Associated clinical syndrome	MODS; cytokine storm; SIRS	Hemorrhagic syndrome
Associate hematologic features		
Schistocytes	Often present	Absent
MAHA/aMAHA	Almost always present	Does not occur
Hepatic coagulopathy	Common	Does not occur
Incidence in clinical practice	Very common	Extremely rare
Management		
Platelet transfusion	Contraindicated	May be used if needed for APL
Treatment	TPE; rADAMTS13 (expected to be very effective)	Treat underlying pathology (e.g., ATRA in APL)

*APL* acute promyelocytic leukemia, *aPTT* activated partial thromboplastin time; *ATRA* All-trans retinoic acid, *DIC* disseminated intravascular coagulation; *DIT* disseminated intravascular microhrombosis; *FDP* fibrin degradation products, *FVIIa* activated factor VII, *FVIII* factor VIII; *MAHA/aMAHA* microangiopathic hemolytic anemia/atypical MAHA, *MODS*, multi-organ dysfunction syndrome, *PT*, prothrombin time; *rADAMTS13* recombinant ADAMTS13, *SIRS* systemic inflammatory response syndrome, *TF* tissue factor, *TMA* thrombotic microangiopathy; *TPE* therapeutic plasma exchange; *TTP* thrombotic thrombocytopenic purpura, *ULVWF* unusually large von Willebrand factor multimers; *VMTD* vascular microthrombotic disease

#### Pathologic coagulation (DVT) vs. microthrombogenesis (DIT)

A very important question is: “where does “DIC” belong to DVT, DIT, or true DIC of APL?” Is DIT conveniently ignored from the standpoint of hemostatic disorder?

In clinical medicine, the physiological mechanism of hemostasis and pathological mechanism of thrombosis has been considered to be the result of the same coagulation process with two different outcomes due to the different circumstance of the injury. Hemostasis is a normal protective physiological process to stop bleeding following external bodily injury, but pathologic thrombosis is the result of normal hemostatic process within intravascular space following intravascular injury [91]. Following TF-activated coagulation cascade, the nature of formed intravascular thrombus (e.g., deep vein thrombosis [DVT] and “DIC”) has been presumed to be the same nature to the hemostatic plug (e.g., blood clots after an injury) [90, 91]. If this is the case, how can we explain that DVT is made of macrothrombus but “DIC” is made of microthrombi? Coagulation scientists have not answered this thought provoking question yet.

Intravascular microthrombosis occurring in “DIC” has been interpreted to be a pathological coagulation disorder mediated through TF-initiated FVII activation [90, 91]. This conception has been strengthened on the following grounds: 1) the term “DIC”, coined by Donald McKay [91, 92], clearly implied that it is a coagulation disorder and this assumption has been accepted by clinicians and coagulation scientists without laboratory and molecular verification, and 2) unlike DIT (i.e., TTP-like syndrome), “DIC” sometimes has occurred with severe hemorrhagic disorder associated with abnormal coagulation profile of prolonged prothrombin time, activated partial thromboplastin time, hypofibinogenemia, and increased fibrin degradation products, which is consistent with consumption of coagulation factors following TF-initiated coagulation cascade. But then, there is chronic “DIC”, which is exactly the same to the feature of endotheliopathy-associated DIT.

However, this abnormal coagulation profile in acute “DIC” is non-specific even though the prevailing interpretation has blamed it to the consumption of coagulation factors in “DIC”. This author differs from this assumption, which will be further discussed later, along with hepatic coagulopathy.

The concept of microthrombogenesis clearly supports that “microthrombi” in DIT are exclusively composed of platelet-ULVWF complexes [1, 2, 4–6]. In contrast, the ironclad concept of “microthrombi” in “DIC” has been “micro blood clots” made of fibrin clots with participation of platelets via TF-initiated coagulation cascade [89–91].

In fact, chronic “DIC” and endotheliopathy-associated DIT (i.e., TTP-like syndrome) are exactly the same in their underlying risk factors, pathologic and phenotypic presentation. First, clinically both disorders almost always occur in critical illnesses; second, their pathology is characterized by arteriolar and capillary hyaline microthrombi with variable fibroblastic proliferation [4, 92]; third, hematologic features are consumptive thrombocytopenia and MAHA. Thus, chronic “DIC” and endotheliopathy-associated DIT are one disease. Until now “DIC” has been incorrectly ascribed to pathological coagulation disorder initiated by TF-induced coagulation [89, 93–97]. This misconception of “DIC” has contributed to many unexplainable mysterious features of “DIC” to date. Now, it is clear that macrothrombus of DVT and microthrombi of DIT/“DIC” occur in intravascular injury due to dissimilar hemostatic pathogenesis.

#### “DIC” perplexity discussed

Table 3 is self-explanatory showing the difference in hematologic and clinical characteristics between endotheliopaathy-associated DIT (i.e., TTP-like syndrome, including McKay’s “DIC”) and true DIC of fibrin clots occurring in APL. In contrast to “DIC”(i.e., false DIC), the predominant feature of true DIC (i.e., in APL) is always hemorrhagic disorder without microvascular thrombosis, MAHA and MODS [87, 98–101]. In regard to “DIC” mystery, a few more comments are appropriate.

First, the International Society on Thrombosis and Hemostasis (ISTH) has introduced the “DIC” scoring system to better establish the diagnosis of “DIC” [102]. It has not been used as a primary diagnostic tool, but has been applied to confirm the diagnosis using hematologic parameters only after predetermined as “DIC” with a clinical disorder known to cause “DIC”. The scoring system has shown low specificity [102]. It should be emphasized that no single laboratory test or set of tests is sensitive or specific enough to allow a definitive diagnosis of “DIC” [103]. In most cases, the diagnosis is based on the combination of results of non-specific abnormal coagulation profile with a clinical condition known to be associated with “DIC” [104].

Second, coagulation scientists put their efforts to support the role of TF in “DIC”, by proposing TF encryption/decryption theory [105], thiol path TF regulation theory [106], TF transfer hypothesis [107], and inflammation and coagulation interaction theory [108–111], which are all still controversial and have not proven the role of TF in “DIC”. Furthermore, the negligible amount of *in vivo* TF, even available, cannot explain the “DIC” presenting with extensive vascular microthrombosis and MODS.

Third, the abnormal coagulation profile, showing prolonged prothrombin time and activated partial thromboplastin time, hypofibinogenemia, and increased fibrin degradation products, is non-specific, and cannot affirm the diagnosis of true DIC. Additionally, this profile oddly develops only in some patients with “DIC”. Therefore, the “chronic/compensated/covert” concept [102, 112, 113], including “low grade DIC”, has been introduced if the coagulation profile is normal or mildly abnormal in “DIC”. This description also cannot explain inexplicably extensive microthrombosis in the absence of depleted coagulation factors.

Fourth, numerous randomized clinical trials (e.g., TF pathway inhibitor, activated protein C, anticoagulant, anti-inflammatory cytokines, and others) to modulate septic response to infection, which pathogenesis is firmly based on interaction theory between inflammation and TF-initiated coagulation, have not been successful to improve the survival in sepsis [114, 115].

It is no wonder, after extensive laboratory studies and clinical trials, why a specific diagnostic test(s) has not been established and no effective treatment discovered for “DIC” after more than half century since DIC was coined in 1950 [91, 92]. The simple answer is that the thesis of TF-initiated pathogenesis of “DIC” has been erroneous.

#### Acute “DIC” is due to DIT-associated hepatic coagulopathy

One remaining, very pertinent question is: “what is the correct diagnosis for acute/decompensated/overt “DIC” that is associated with abnormal coagulation profile?” The answer is clear since “DIC” is often associated with hepatic diseases [1, 15, 116–120]. In endotheliopathy-associated DIT, hepatic coagulopathy could occur as a phenotype of acute fulminant hepatic failure or acute hepatic necrosis as seen in critical illnesses [15, 90, 116–120]. The pathogenesis unknown hepato-renal syndrome [121–123] and hepatic encephalopathy [15, 121] are very much consistent with endotheliopathy-associated DIT similar to “DIC” syndrome. Thus, acute “DIC” is hepatic coagulopathy occurring in endotheliopathy-associated DIT.

Indeed, the medical literature is replete with DIT, “DIC” and/or hepatic failure, occurring in association with HELLP syndrome, HUS, acute necrotizing pancreatitis, purpura fulminans, rhabdomyolysis, acute respiratory distress syndrome, viral hemorrhagic fevers as well as hepato-renal syndrome and hepatic encephalopathy. In retrospect, when clinical phenotype of acute fulminant hepatic failure or acute hepatic necrosis presents with thrombocytopenia and coagulopathy, Enthotheliopathy-associated DIT (i.e., TTP-like syndrome) should be suspected rather than the diagnosis of acute “DIC” [26]. Indeed, hepatic coagulopathy occurring in DIT is a life-threatening thrombo-hemorrhagic syndrome without correct diagnosis [26].

#### Differential diagnosis of true DIC and TTP-like syndrome

In differentiating true DIC (e.g., APL) from DIT with hepatic coagulopathy, the most reliable test is the assay of coagulation factors [1, 2, 87, 99, 124, 125] (Table 4). In true DIC, FVIII and FV are markedly decreased due to their consumption and inactivation, but, in DIT with hepatic coagulopathy (i.e., acute “DIC”), FVIII is normal or more likely markedly increased and FVII is markedly decreased. The increase of FVIII in hepatic coagulopathy is likely due to endothelial exocytosis of ULVWF, in which some of ULVWF are cleaved by ADAMTS13 to smaller VWF multimers and released into circulation to bind FVIII and protect it from degradation. A suggested guideline for laboratory tests is summarized in Table 4 to aid in the differential diagnosis among complicated thrombopathies and coagulopathies [1, 2].

**Table 4:** Hematologic differential diagnoses among thrombopathies and coagulopathies

	TTP & TTP-like syndrome (DIT)	TTP-like syndrome (DIT) associated with HC (e.g., sepsis) equal to acute “DIC”	DIC (e.g., APL)	PF (e.g., amyloidosis)
Thrombocytopenia	Always present	Always present	Present due to APL, but not due to consumption (?)	Not present
MAHA/aMAHA	Always present	Always present	Do not occur	Not present
Fibrinogen	Normal	Decreased	Always decreased	Always decreased
Factor VIII	Normal	Normal or increased	Markedly decreased	Decreased
Factor V	Normal	Decreased	Decreased	Normal or decreased
Factor X	Normal	Decreased	Usually normal	Normal (?)
Factor VII	Normal	Markedly decreased	Normal	Normal
Factor IX	Normal	Decreased	Normal	Normal
FDP	Normal	?	Positive	Strongly positive
Prothrombin time	Normal	Prolonged	Prolonged	Prolonged
Activated partial thromboplastin time	Normal	Prolonged	Prolonged	Prolonged
Thrombin time	Normal	Prolonged	Prolonged	Prolonged
Thrombosis form	Microthrombi	Microthrombi	Friable fibrin clots (meshes)	Absent
Bleeding: Character Treatment	Petechiae; Usually no need of treatment	May cause serious bleeding; Controllable with FFP & rFVIIa	Common, serious bleeding; Abrogated with ATRA & chemotherapy	Slow & persistent bleeding; Treatable with AFA
Hypoxic organ dysfunction (MODS)	Present	Present	Not present	Not present
Platelet transfusion	Contraindicated	Contraindicated	May be used for APL	Not needed

*AFA* anti-fibrinolytic agent, *ATRA* all-trans retinoic acid, “*DIC*” false disseminated intravascular coagulation, *DIT* disseminated intravascular microthrombosis, *FDP* fibrin degradation products, *FFP* fresh frozen plasma; *HC* hepatic coagulopathy; *MAHA/aMAHA* microangiopathic hemolytic anemia/atypical MAHA, *PF* primary fibrinolysis, *TTP* thrombotic thrombocytopenic purpura

### Hope for the future in the treatment

VMTD presenting with both TTP and TTP-like syndrome has responded well to TPE if the treatment is initiated in the earliest possible stage [11–13, 20, 41, 42]. In medical literature, there is a plethora of case reports of successful treatment with TPE for atypical TTP and TTP-like syndrome. Once the disease progresses to the point of irreversible organ dysfunction due to tissue hypoxia, recovery is unlikely to occur in TTP-like syndrome [13]. This is particularly true in ARDS [20], encephalopathy in HUS [126], and adrenal insufficiency in sepsis (i.e., septic shock) [127].

High mortality associated with “DIC” in critically ill patients could have been related to platelet transfusions as well as masked hepatic coagulopathy and heparin treatment. The platelet transfusion is contraindicated because it aggravates on-going microthrombogenesis, and heparin treatment increases hemorrhage in hepatic coagulopathy. TPE is the treatment of choice for DIT at this time. Fresh frozen plasma or recombinant FVIIa [128] to replace the lowest FVII might have been beneficial for severe hemorrhage in hepatic coagulopathy associated with VMTD.

Theoretically, two very promising targeted therapeutic approaches for TTP-like syndrome are 1) anti-complement therapy such as eculizumab and recombinant CD59 to inhibit the first leg of the pathogenesis based on “two-activation theory of the endothelium” [3, 129–131] and 2) anti-microthrombotic therapy such as recombinant ADAMTS13 to correct or modify the second leg of the endothelial pathogenesis [3]. Indeed, eculizumab has shown promising results [129, 130]. However, anti-complement therapy should be explored with an extreme care since it could cause catastrophic harm in the septic patient by aggravating the ongoing process of sepsis and septic shock. Currently, recombinant ADAMTS13 is being investigated for the treatment of hereditary TTP. Since it has shown to cleave eULVWF [4, 6, 69], controlled clinical trials should be initiated for TTP-like syndrome as soon as possible. If it is effective, recombinant ADAMTS13 could save so many lives, especially in the critical care medicine, obstetrics, surgery, transplant and more.

## Conclusion

TTP and TTP-like syndrome are two different diseases caused by dissimilar pathogenesis although their underlying pathologic feature of DIT and hematologic phenotype are similar. TTP is intravascular microthrombotic disease due to ADAMTS13 deficiency, but TTP-like syndrome is hemostatic disease associated with endotheliopathy in critical illnesses. It is essential to recognize TTP-like syndrome as a distinct disease entity, which working diagnostic criteria is summarized in Table 5. These criteria would benefit the patient through earlier unmasking of the diagnosis and life-saving TPE when presented with atypical organ phenotypic syndromes. Certainly, “two activation theory of the endothelium” has been able to clarify many unresolved issues of thrombotic microangiopathy. Among them are TTP, HUS, TTP-like syndrome, MODS, “DIC” and combined organ dysfunction syndrome with hepatic coagulopathy.

**Table 5:** Proposed working diagnostic criteria for TTP-like syndrome

1. Thrombocytopenia and MAHA/aMAHA.	
2. Underling critical illness due to such conditions as.	
**√** Pathogen (bacterial; viral; fungal; rickettsial; parasitic)	
**√** Polytrauma (chest and lung; bone; skull/brain)	
**√** Pregnancy (preeclampsia; abruptio placenta; amniotic fluid embolism)	
**√** Cancer (breast; stomach; lung)	
**√** Surgery (heart; bowel; uterus; bone)	
**√** Transplant (liver; kidney; bone marrow)	
**√** Disease (autoimmune vascular disease; malignant hypertension)	
**√** Drug (cyclosporin; mitomycin C)	
**√** Toxin (venom; ricin; Shiga toxin)	
3. Negative antibody against ADAMTS13.	
4. Mild to moderately decreased activity of ADAMTS13 (20–70% of normal).	
5. One or more organ phenotype dysfunction syndromes such as.	
**√** Pancreatitis.	
**√** Myocardial infarction.	
**√** ARDS.	
**√** Acute fulminant hepatic failure.	
**√** Acute adrenal insufficiency.	
**√** Rhabdomyolysis.	
**√** Non-occlusive mesenteric ischemia.	
**√** Hepato-renal syndrome.	
**√** Hepatic-encephalopathy.	
**√** Cardio-pulmonary syndrome.	
**√** Tissue gangrene.	
**√** Peripheral digit ischemic syndrome.	

+ Encephalopathy and ARF are common in both TTP and TTP-like syndrome

*ARDS* acute respiratory distress syndrome, *TTP* thrombotic thrombocytopenic purpura

Both activation of complement through C5b-9 in critical illnesses and microthrombogenesis with participation of the platelet and ULVWF have unequivocally supported the crucial role of endotheliopathy in the pathogenesis of TTP-like syndrome. The role of endothelial protectin CD59 and C5b-9 should be evaluated to further support the endothelial molecular pathogenesis in TTP-like syndrome [131]. The future therapeutic modalities should be explored with anti-complement therapy and anti-microthrombotic therapy. This author believes anti-microthrombotic therapy is safer option.

Lastly, the designation of the term VMTD is very appropriate not only for TTP and TTP-like syndrome, but also to include hereditary focal, multifocal and localized microthrombotic diseases, and acquired microthrombotic disorders such as stroke syndromes, cardiac angina, and coronary procedure-associated transient microthrombotic syndrome. The recognition of the term VMTD would further assist in the understanding of endothelial physiology and identifying of endothelial pathophysiology in many human diseases, especially in hemostasis and hemostatic disorders.

## Abbreviations

ADAMTS13: A disintegrin and metalloproteinase with a thrombospondin type 1 motif, member 13
AFA: Anti-fibrinolytic agent
AFHF: Acute fulminant hepatic failure
AI: Adrenal insufficiency
APL: Acute promyelocytic leukemia
aPTT: Activated partial thromboplastin time
ARDS: Acute respiratory distress syndrome
rADAMTS13: Recombinant ADAMTS13
ARF: Acute renal failure
ATRA: All-trans retinoic acid
DIC: Disseminated intravascular coagulation
“DIC”: false disseminated intravascular coagulation
DIT: Disseminated intravascular thrombosis
ECs: Endothelial cells
FDP: Fibrin degradation products
FFP: Fresh frozen plasma
FVIIa: Activated factor VII
FVIII: Factor VIII
HC: Hepatic coagulopathy
IL: Interleukin
HELLPs: Hemolysis elevated liver enzymes and low platelet syndrome
HUS: Hemolytic uremic syndrome
LDH: Lactate dehydrogenase
MAC: Membrane attack complex
MI: Myocardial infarction
MAHA: Microangiopathic hemolytic anemia
aMAHA: Atypical MAHA
MODS: Multi-organ dysfunction syndrome
PF: Purpura fulminans
PT: Prothrombin time
SIRS: Systemic inflammatory response syndrome
SS: Stroke syndrome
STEC: Shiga toxin producing *E. coli*
TCIP: Thrombocytopenia in critically ill patient
TF: Tissue factor
TPE: Therapeutic plasma exchange
TTP: Thrombotic thrombocytopenic purpura
vWF: von Willebrand factor
ULVWF: Unusually large von Willebrand factor multimers
eULVWF: Endothelial ULVWF
mULVWF: Megakaryocytic ULVWF
VMTD: Vascular microthrombotic disease
AA-VMTD: antibody associated VMTD
EA-VMTD: Endotheliopathy associated VMTD
GA-VMTD: Gene mutation associated VMTD
